# A Case of Extramedullary Plasmacytoma of the Biliary Tract with a Poor Prognosis

**DOI:** 10.3390/reports6010001

**Published:** 2022-12-29

**Authors:** Eiko Sakurai, Kazunori Nakaoka, Seiji Yamada, Naoe Goto, Akihiro Tomita, Yoshiki Hirooka, Tetsuya Tsukamoto

**Affiliations:** 1 Department of Diagnostic Pathology, Graduate School of Medicine, Fujita Health University, 1–98, Dengakugakubo, Kutsukake-cho, Toyoake 470-1192, Japan; 2 Department of Gastroenterology, Graduate School of Medicine, Fujita Health University, 1–98, Dengakugakubo, Kutsukake-cho, Toyoake 470-1192, Japan; 3 Department of Hematology, Graduate School of Medicine, Fujita Health University, 1–98, Dengakugakubo, Kutsukake-cho, Toyoake 470-1192, Japan

**Keywords:** plasmacytoma, solitary plasmacytoma, extraosseous plasmacytoma, extramedullary plasmacytoma, bile duct, gall bladder

## Abstract

Extramedullary plasmacytoma (EMP) is a rare disease consisting of the presence of monoclonal plasma cells in tissues other than the bone. Most EMPs are located in the head and neck region. We present an extremely rare case of an EMP originating from the biliary tract in a 76-year-old male. This is the fifth report of a primary EMP arising from the biliary tract. He was diagnosed with jaundice, and he was referred for an additional examination. Abdominal ultrasonography revealed a tumor in the gallbladder and bile ducts, and a bile duct biopsy was performed via endoscopic ultrasound-guided fine-needle aspiration (EUS-FNA). The pathological and immunohistochemical examination revealed that the tumor was a plasmacytoma originating in the biliary tract. Although endoscopic biliary drainage was performed, the bile duct infection was not well controlled due to obstructive jaundice caused by the tumor. Furthermore, the bleeding from the tumor during chemotherapy was uncontrolled. Pancreaticoduodenectomy and cholecystectomy were performed to control the infection and bleeding. Although chemotherapy was continued after surgery, the tumor of the intrahepatic bile duct enlarged. He died seven months after the diagnosis because of the treatment-resistant tumor.

## 1. Introduction

Plasma cell neoplasms are characterized by increased monoclonal immunoglobulin secretion. These neoplasms can arise in either multiple lesions (multiple myeloma (MM)) or a single lesion (solitary plasmacytoma). Solitary plasmacytoma can be localized in the bone (solitary plasmacytoma of the bone (SPB)) or in other tissues (extramedullary (extraosseous) plasmacytoma (EMP)). EMP, a rare disease, accounts for 1–3% of all plasma cell proliferation [1]. In approximately 80% of the cases, the EMPs are located in the upper airways, such as in the head and neck region [2,3], while the other locations are the gastrointestinal tract, lymph nodes, bladder, breast, thyroid, testes, and parotid glands [3]. There have been only four reports of primary extramedullary plasmacytoma arising from the biliary tract [4,5,6,7] and five reports of secondary bile duct plasmacytoma with MM [8,9,10,11,12]. An EMP occurs predominantly in males, with the average age at onset being from 55 to 60 years [13]. Dores, G.M et al. [14] reported a significant incidence of EMPs in Black men. Being a younger age at the onset of the EMP results in a better prognosis [13,15].

The EMP does not have apparent clinical features, while MM expresses systematic organ damage, such as hypercalcemia, renal insufficiency, anemia, and bone lesions. The symptoms of EMP depend on the location of the tumor. Approximately 20% of EMP patients have an M protein, which is mostly composed of IgA [3]. The prognosis of an EMP is relatively good compared to that of MM, the extramedullary infiltration of MM, and plasmablastic lymphoma [3,8]. The progression to MM occurs in approximately 15–30% of EMP cases, resulting in worse clinical outcomes [3,16]. The morphological features of EMPs include the proliferation of monoclonal plasma cells, such as MM and SPB. The differential diagnoses of this condition are extranodal marginal zone lymphoma (MZL) of the mucosa-associated lymphoid tissue (MALT lymphoma), plasmablastic lymphoma, and extraosseous infiltrates of MM.

## 2. Case Presentation

**Figure 1:**
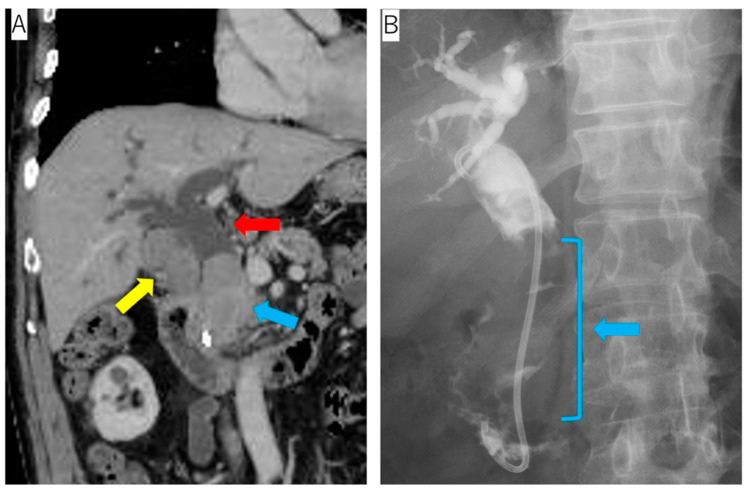
Radiological evaluation. (**A**) Contrast-enhanced CT revealed an ischemic tumor of the gallbladder (yellow arrow) and common bile duct (blue arrow) as well as common bile duct dilation (red arrow). (**B**) ERCP revealed an expansive tumor in the common bile duct.

A 76-year-old man presented with jaundice during treatment for hypertension, diabetes, and hyperuricemia. Abdominal ultrasonography revealed a solid tumor in the gallbladder and common bile duct dilatation. Contrast-enhanced computed tomography (CT) detected an ischemic tumor of the gallbladder and the common bile duct and the dilation of the common bile duct. Endoscopic retrograde cholangiopancreatography (ERCP) revealed an expansive tumor in the common bile duct (Figure 1).

The blood test findings indicated hyperbilirubinemia, mild anemia, high CRP levels, and high IL-2R levels. CEA and CA19-9 were within the normal limits. High IgA titers were also observed (Table 1).

The bile cytology revealed the proliferation of degenerated atypical cells with poor epithelial cohesiveness. The immunohistochemical analyses of the cell blocks from the bile cytology specimens expressed positivity for CD45 LCA and vimentin and negativity for cytokeratin (CK) (AE1/AE3), CD3, CD20, CD79a, and CD30. Although a hematopoietic tumor was suspected, the details could not be determined.

After these tests were performed, endoscopic ultrasound-guided fine-needle aspiration (EUS-FNA) for the bile duct tumor was performed. The proliferation of the monoclonal atypical cells was observed with hematoxylin and eosin (H&E) staining. Immunohistochemically, these cells were positive for CD138 and negative for CD3, CD20, CD79a, and CK (AE1/AE3). The κ light chain was positive, while the λ one was negative. Genetic and chromosomal analyses for the EMP were not performed. The subsequent examination revealed no bone marrow involvement. Therefore, the diagnosis of a primary EMP localized in the biliary tract was confirmed.

The patient was administered bortezomib, dexamethasone, and lenalidomide (BLd). However, lenalidomide was discontinued because of the erythroderma that occurred as a severe adverse event after 7 days of administration, and the patient instead received three courses of a bortezomib and dexamethasone (Bd) regimen. The patient had diabetes mellitus as a comorbidity, and he required insulin therapy with dexamethasone, which forced him to reduce the dose of dexamethasone. Despite endoscopic biliary drainage being performed, it was difficult to control the biliary tract infection associated with obstructive jaundice due to the tumor. Bleeding from the tumor also occurred. After four rounds of chemotherapy, he underwent pancreaticoduodenectomy and cholecystectomy to control repeated cholangitis and bleeding from the bile ducts.

**Figure 2:**
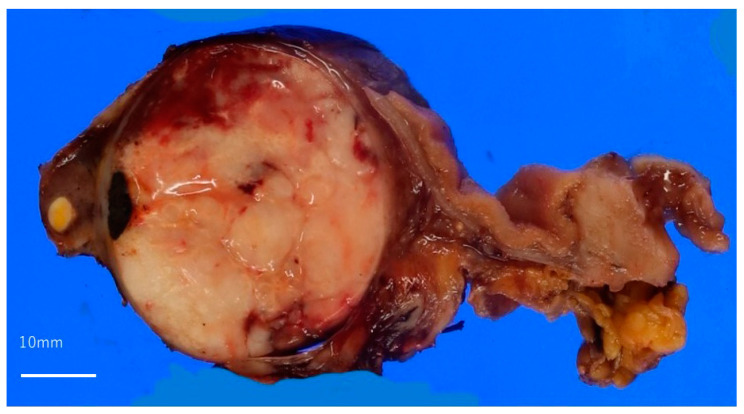
Macroscopic view of a resected bile duct. A whitish solid tumor is filling the common bile duct.

**Figure 3:**
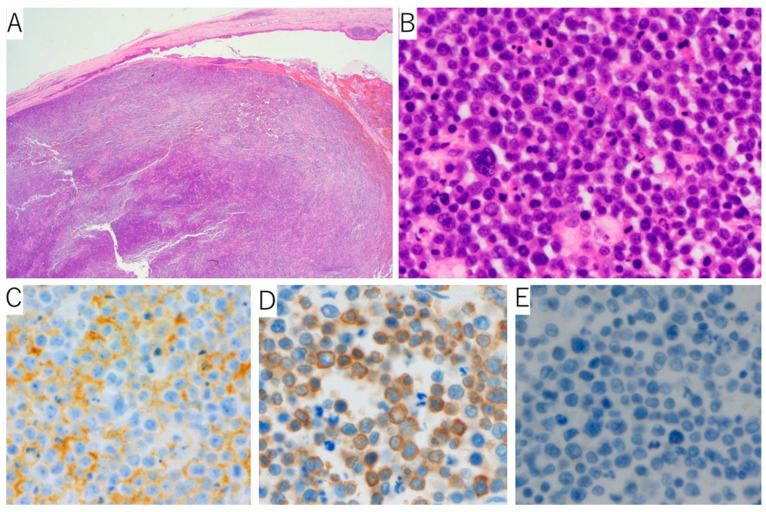
Microscopic view of the bile duct tumor: (**A**) The tumor fills the bile duct. H&E staining, original magnification, ×12.5. (**B**) Magnified view, H&E, ×400. (**C**) CD138 immunostaining, ×400. (**D**) The immunoglobulin k light chain in situ hybridization, ×400. (**E**) The l light chain in situ hybridization, ×400.

A whitish solid tumor filling in the bile duct and gallbladder was observed (Figure 2). In the H&E sections, the monoclonal cells that looked like plasma cells were proliferating. The immunohistochemical staining revealed CD138 positivity, and immunoglobulin κ light chain *in situ* hybridization was positive for most of the plasma cells, whereas that of the λ chain was negative (Figure 3). After the surgery, the tumor’s progression rapidly became more similar to plasmablastic lymphoma, so systemic therapy, including cyclophosphamide, doxorubicin, vincristine, prednisone, and etoposide (EPOCH), was performed; however, the patient died of tumor bleeding, febrile neutropenia, and severe cholangitis after two cycles of the EPOCH regimen.

## 3. Discussion

EMP must be distinguished from an extranodal MZL of the MALT lymphoma, extraosseous infiltrates of MM, and plasmablastic lymphoma. MALT lymphoma is a low-grade B-cell lymphoma with prominent plasmacytic differentiation. A well-known pathological feature of MALT lymphoma is the presence of a lymphoepithelial lesion (LEL), which is the lymphocytic infiltration of the epithelial cells. LEL was not evident in this case. MM was ruled out because no bone marrow lesions were detectable and there were no clinical features of MM. Plasmablastic lymphoma commonly occurs in immunocompetent patients due to human immunodeficiency virus infection and iatrogenic immunosuppression. The EBV infection is strongly associated with plasmablastic lymphoma. In this case, the *in situ* hybridization of EBV-encoded RNA and CD56 were negative, which is unlike typical cases of plasmablastic lymphoma. Although the negative immunoexpression of CD20 and CD79a caused difficulty in making a histological diagnosis, positive CD45 LCA staining indicated hematopoietic malignancy. The final diagnosis was made according to the CD138 expression as well as immunoglobulin k light chain restriction.

The prognosis of an EMP is relatively good overall. Some case reports have mentioned the prognosis of primary EMP arising from the gall bladder. Kondo et al. reported a 53-year-old male who was confirmed to be alive for three years and seven months after being diagnosed [4]. Hwang et al. described the case of a 63-year-old female patient with no evidence of the disease for eight years after surgery [5]. Majerović et al. reported the case of a 69-year-old man who showed no sign of recurrence for two years [6]. In contrast to these reports, the patient in this case had a poor prognosis as he survived for only seven months after the diagnosis was made. The diagnosis of the patient in this case report needed to be distinguished from plasmablastic lymphoma and extraosseous infiltrates of MM, which are both associated with aggressive courses. Clinically and per the immunohistochemistry, these diseases were excluded, as mentioned above.

Hughes et al. [17] discussed the differences in the molecule patterns between 14 solitary plasmacytomas (four EMP and ten SPB) and 11 MMs. CD49d (Integrin α4), which are related to the promotion of plasma cell colonization, the prevention of cell apoptosis, and the development of cytotoxin resistance, and they showed a statistically significant difference between MM and solitary plasmacytomas. From this report, it might be suggestive that MM shows a poorer prognosis compared to EMP because the CD49d expression in MM was higher than that in EMP.

Although there are several reports which discuss the better prognosis of EMPs compared to MMs [3,15,18], this case did not show a favorable prognosis. The prognosis of EMP usually worsens when it progresses to MM. In this case, the prognosis became poorer because the tumor progression was aggressive in a similar way to plasmablastic lymphoma. It is rare to have a poor prognosis without progression to MM.

Radiation therapy or surgery is generally the treatment of choice for localized solitary extramedullary plasmacytoma, but because the area occupied by the EMP extended into the intrahepatic bilateral bile ducts, gallbladder, and common bile duct, the irradiated area was considered to extend more into the liver, and radiation therapy was not chosen because of the high risk of severe liver failure and cholangitis. Pancreatoduodenectomy was forced to be chosen to control the local infection and bleeding, although it was a very strong invasive therapy.

Although immunotherapy with CD38-targeting antibodies might be an effective approach, this treatment was not covered as an induction therapy for MM by the national insurance in Japan at that time. Thus, the patient could not receive therapy using CD38-targeting antibodies.

## 4. Conclusions

EMP is difficult to diagnose because it’s symptoms vary with the organ from which the tumor originates. However, EMP must not be misdiagnosed as other plasma cell neoplasia such as extraosseous infiltrates of MM, plasmablastic lymphoma, or lymphomas with plasmacytic differentiation such as MALT lymphoma since their treatments and prognoses are totally different from those of EMP.

## Figures and Tables

**Table 1:** Blood chemistry.

Test Item	Measured Value	(Normal)	Unit	Test Item	Measured Value	(Normal)	Unit
WBC	8000	(3.3–8.6)	/μL	AST	88	(13–30)	U/L
RBC	3.58 × 10^6^	(4.35–5.55)	/μL	ALT	139	(10–42)	U/L
Hb	11.7	(13.7–16.8)	g/dL	γ-GT	1177	(13–64)	U/L
Plt	38.5 × 10^4^	(15.8–34.8)	/μL	Cre	1.3	(0.65–1.07)	mg/dL
TP	7.2	(6.6–8.1)	g/dL	BUN	30.7	(8–20)	mg/dL
Alb	2.9	(4.1–5.1)	g/dL	CRP	8.6	(0.00–0.14)	mg/dL
IgA	794	(93–393)	mg/dL	AFP	3.9	(0.0–10.0)	ng/mL
T-Bil	6.3	(0.4–1.5)	mg/dL	IL-2R	1090	(204.5–587.3)	U/mL
D-Bil	5.2	(0.0–0.4)	mg/dL	CEA	<1.7	(0.0–5.0)	ng/mL
ALP	837	(38–113)	U/L	CA19-9	20.3	(0.0–37.0)	U/mL

## Data Availability

The data used in this case report are available upon reasonable request from the corresponding author.

## References

[1] Him M., Meier M., Mehta V. (2017). Rare Presentation of Primary Extramedullary Plasmacytoma as Lip Lesion. Case Rep. Oncol. Med..

[2] Galieni P., Cavo M., Pulsoni A., Avvisati G., Bigazzi C., Neri S., Caliceti U., Benni M., Ronconi S., Lauria F. (2000). Clinical outcome of extramedullary plasmacytoma. Haematologica.

[3] McKenna R.W., Kyle R.A., Kuehl W.M., Harris N.L., Coupland R.W., Fend F., Swerdlow S.H., Campo E., Harris N.L., Jaffe E.S., Pileri S.A., Stein H., Thiele J. (2017). Plasma cell neoplasms. WHO Classification of Tumours of Haematopoietic and Lymphoid Tissues.

[4] Kondo H., Kainuma O., Itami J., Minoyama A., Nakada H. (1995). Extramedullary plasmacytoma of maxillary sinus with later involvement of the gall bladder and subcutaneous tissues. Clin. Oncol. J. (R. Coll. Radiol.).

[5] Hwang D.W., Lim C.S., Jang J.Y., Lee S.E., Yoon S.O., Jeon Y.K., Uk Lee K., Kim S.W. (2010). Primary hematolymphoid malignancies involving the extrahepatic bile duct or gallbladder. Leuk. Lymphoma.

[6] Majerović M., Bogdanić B., Drinković N., Kinda S.B., Jakić-Razumović J., Gasparović V. (2012). Extramedullary plasmacytoma imitating neoplasm of the gallbladder fossa after cholecystectomy. Coll. Antropol..

[7] Schuster D., Klosterhalfen B., Fiedler C., Prescher A. (2007). Metastasis of medullary plasmocytoma as the cause of acute cholecystitis. Dtsch. Med. Wochenschr..

[8] St Romain P., Desai S., Bean S., Jiang X., Burbridge R.A. (2015). Extramedullary plasmacytoma of the gallbladder diagnosed by endoscopic ultrasound fine needle aspiration (EUS-FNA). J. Gastrointest. Oncol..

[9] Heckmann M., Uder M., Grgic A., Adrian N., Bautz W., Heinrich M. (2008). Extraosseous manifestation of multiple myeloma with unusual appearance in computed tomography--case report. Rontgenpraxis.

[10] Fukatsu H., Hiramatsu Y., Takagi S., Morishita H. (2013). Multiple myeloma involving the extrahepatic bile duct. Intern. Med..

[11] Abughanimeh O., Qasrawi A., Abu Omar M., Bahaj W., Abu Ghanimeh M. (2018). A Case of Multiple Myeloma Associated with Extramedullary Plasmacytoma of the Gallbladder Manifesting as Acute Cholecystitis. Cureus.

[12] Abt A.B., Deppisch L.M. (1969). Multiple myeloma involving the extrahepatic biliary system. J. Mt. Sinai. Hosp. N. Y..

[13] Lai C.Y., Hsieh H.H., Chen H.K., Chao C.Y., Hua C.H., Tai C.J., Bau D.T., Tsai M.H., Shih L.C. (2020). Clinical Features of Head and Neck Solitary Extramedullary Plasmacytoma in Taiwan. In Vivo.

[14] Dores G.M., Landgren O., McGlynn K.A., Curtis R.E., Linet M.S., Devesa S.S. (2009). Plasmacytoma of bone, extramedullary plasmacytoma, and multiple myeloma: Incidence and survival in the United States, 1992–2004. Br. J. Haematol..

[15] Shih L.Y., Dunn P., Leung W.M., Chen W.J., Wang P.N. (1995). Localised plasmacytomas in Taiwan: Comparison between extramedullary plasmacytoma and solitary plasmacytoma of bone. Br. J. Cancer.

[16] Merza H., Sarkar R. (2016). Solitary extraosseous plasmacytoma. Clin. Case Rep..

[17] Hughes M., Doig A., Soutar R. (2007). Solitary plasmacytoma and multiple myeloma: Adhesion molecule and chemokine receptor expression patterns. Br. J. Haematol..

[18] Goyal G., Bartley A.C., Funni S., Inselman J., Shah N.D., Marshall A.L., Ashrani A.A., Kapoor P., Durani U., Hashmi S.K. (2018). Treatment approaches and outcomes in plasmacytomas: Analysis using a national dataset. Leukemia.

